# The (failed) promise of multimorbidity: chronicity, biomedical categories, and public health

**DOI:** 10.1080/09581596.2021.2017854

**Published:** 2021-12-30

**Authors:** Rebecca Lynch, Benjamin Hanckel, Judith Green

**Affiliations:** a Department of Women & Children's Health, King's College London, London, UK; b Western Sydney University Institute for Culture and Society, Penrith South, Australia; c University of Exeter, Wellcome Centre for Cultures and Environments of Health, Exeter, United Kingdom of Great Britain and Northern Ireland

**Keywords:** Multimorbidity, chronicity, situational analysis, public health

## Abstract

Multimorbidity has become an increasingly prominent lens through which public health focuses on the ‘burden’ of ill health in ageing populations, with the promise of a more upstream and holistic approach. We use a situational analysis (drawing on documentary analysis and interviews with service providers, policy actors and people living with multiple conditions) in south London, UK, to explore what this lens brings into focus, and what it obscures. Local initiatives mobilised the concept of multimorbidity in initiatives for integrating health care systems and for commissioning for prevention as well as care. However, as the latest of a series of historical attempts to address system fragmentation, these initiatives generated more complexity, and a system orientated to constant transformation, rather than repair or restoration. Service providers and patients continued to struggle to navigate the system. Dominant policy and practice narratives framed patient self-management as the primary route for addressing individualised risk factors on a trajectory to multimorbidity, whereas the narratives of those living with multiple conditions were more oriented to a relational model of health. The findings suggest possibilities and limitations for leveraging the concept of multimorbidity for public health. In this field, the promise arose from its potential to make spaces for a focus on populations, not patients with discrete diseases. Realising this promise, however, was limited by the inherent tensions of biomedical nosologies, which separate discrete diseases within individual bodies, and from epidemiological approaches that reify the socio-material contexts of failing health as risks for individuals.

## Introduction

Multimorbidity has come to the fore in recent years as a way of framing the public health implications of the growing burden of population ill health (Stirland et al., 2020; Xu et al., 2017). Two major rationales given for this interest are the rising number of people identified as simultaneously having two or more (long-term) medical conditions, particularly in the context of ageing populations, and the inequalities associated with being recorded as having two or more conditions (Barnett et al., 2012; Pefoyo et al., 2015; Queen et al., 2017). That is, the public health ‘problem’ is framed primarily as one of the growing social and economic (Soley-Bori et al., 2020) burden of ill health, and its inequitable distribution, with the less advantaged at higher risk.

That multimorbidity can be a problem is, conceptually, an inevitable outcome of contemporary biomedical knowledge organised around ‘diseases’; discrete entities, with their own constellations of pathology, natural history and symptoms. That diseases are separate makes it possible to live with, or suffer from, more than one disease at once. This is not an inevitable feature of medical knowledge. Other medical cosmologies might be, for instance, organised around patients’ sickness, rather than underlying diseases. Jewson’s (1976) formulation of historical changes in medical cosmologies, for example, located the emergence of diseases as the primary object of medical knowledge as an innovation resulting from medicine organising as a profession around hospitals in the late 18^th^ century. Prior to this, he argued, the sick patient ailed with a unique and subjective set of symptoms relating to their indivisible self: to have spoken of ‘multiple pathologies’ within one patient would have been inconceivable. Instead, argued Jewson, pathologies multiplied outside the patient, with a cosmology characterised by a plethora of local, contingent and conflicting medical theories. With a shift in focus from the ‘sick patient’ to the disease (in Jewson’s terms), came the possibility of one patient being ill with multiple diseases, which might afflict different organs or systems of the body and/or the mind. For biomedicine, these multiple diseases were typically termed ‘comorbidities’. This term has increasingly been replaced with ‘multimorbidities’ to reflect the lack of primacy of any one disease (Van Den Akker et al., 1996). Over the last two decades, multimorbidity has increasing become a global research priority (Academy of Medical Sciences (AMS), 2018; Xu et al., 2017), with its own separate MeSH (Medical Subject Heading) term since 2018 (Nicholson et al., 2019).

There are, however, a number of challenges for this relatively new field of epidemiological research. Health service records and mortality statistics are organised primarily around discrete disease categories, and studying multimorbidity entails first constructing a new category. If broadly viewed as ‘more-than-one long-term condition’ (Fortin et al., 2012; Pefoyo et al., 2015), multimorbidity could well characterise much of the population much of the time: the conditions that ‘count’ towards being classified as multiply-morbid need to be restricted to those that matter. Yet delineating which conditions matter, and to whom, is not straightforward (Lefèvre et al., 2014). One review (Fortin et al., 2012) noted the variability in the number of diseases which would ‘count’ across published prevalence studies, with some studies including any diagnosis of a long-term condition, with no regard to prevalence of contributing condition, severity or impact on wellbeing, and others including only the most prevalent or health damaging. Whether particular diagnoses count as separate diseases, or manifestations of the same underlying disorder, is also a challenge, given that morbidities tend to cluster (Prados-Torres et al., 2014). Clusters of conditions that are statistically likely to appear together can be conceived of as syndromes of disorder that, rather than representing discrete pathological processes, might instead represent different (symptom) manifestations of the same underlying process (Schäfer et al., 2010). Finally, constructing multimorbidity also brings into sharp relief the well-rehearsed complexities of delineating pathology from normal variation, and causes from outcomes (Canguilhem, 1949/1991, King, 1954). Are hypertension or obesity to count as ‘diseases’ contributing to multimorbidity? Or are they ‘risk factors’ for it – or better conceptualized as outcomes of other diseases, or their management?

If ‘multimorbidity’ is a challenge for epidemiological research, it is also widely acknowledged as a challenge to the practices and technologies of health care built on contemporary biomedical nosology (Banerjee, 2015; Mangin et al., 2012; Whitty et al., 2020). Medicine-as-practice reflects medical knowledge. Professional specialisms are organised around particular bodily systems and diseases, and health care provision is typically materialised in disease-specific clinics. A conceptual shift that brings different conditions together collides with the embedded structures of healthcare provision.

In principle, this collision arising from managing multimorbidity in a system designed around single diseases might be a point of refocusing for health care – away from the hospital and primary care clinics organized around patients with diabetes, hypertension or mental illness, and towards a more holistic framing of health and wellbeing. Mapping the multiplicity of disorders makes visible what Manderson and Warren (2016) call the ‘recursive cascades’ of chronicity in syndemics, in which social and environmental conditions coalesce to make populations vulnerable to ill health. A shift in focus from patients with discrete diseases to people living with multiple morbidities should make possible a more public health focused approach, revealing how conditions are clustered, and which groups of people in which places might be particularly affected (Whitty et al., 2020). The more holistic approach suggested by multimorbidity, with an orientation towards the multiplicity of patients’ experiences, not diseases, and an epidemiological lens focused on population distributions of ill health, potentially opens up a more ‘upstream’ approach, in keeping with a public health agenda for sustaining healthy populations and places.

We aimed to explore what multimorbidity was ‘doing’, as a relatively new concept in healthcare, and with what implications for realizing a more public health-orientated approach to chronicity. Our case was the health system in one setting: two neighbouring boroughs, Southwark and Lambeth, in London, UK. These boroughs have separate local government authorities, public health directorates and commissioning bodies, but with many cross-borough provisions and health and wellbeing initiatives. Between them, the boroughs have a population of around 630,000 people, served by around 85 NHS (National Health Service) GP (General Practitioner) practices providing primary care, two trusts providing in-patient hospital services, and a large number of other NHS health providers.

## Methods

To explore what the concept of multimorbidity was doing in this field, we drew on the techniques of situational analysis (Clarke, 2003). Situational analysis is an approach rooted in grounded theory, but which takes the non-human as well as human elements of a field seriously, focusing on the relational ecologies of a ‘situation’. Methodologically, processes include identifying the major actors (organisations, people, discourses, policies) in a situation, the relationships between them, and the key areas of debate, negotiation and framing of the topic of interest. Situational analysis is therefore an approach particularly suited to fields that are complex and uncertain, and where understandings are unfolding in real time. Our ‘situation’ was the (public) health system across the two boroughs. The changes unfolding in this field were in part prompted by the emerging global focus on multimorbidity, with its calls for research and action. Locally, these had engendered a large programme of work begun in 2017, which this study was part of: The Multiple Long Term Conditions Programme, funded by a local philanthropic organisation. The broader programme was a response to recommendations by a report commissioned by the Richmond Group of voluntary organisations (Aidan 2018) which had called for action on multiple conditions.

We drew on a range of data sources, including: 42 documents related to local health policy and practice; eight formal interviews with local health policy or practice key informants (e.g. commissioning leads, clinical leads); informal interviews and observations (of health care settings, research meetings) and 18 interviews with people (eight men, 10 women) living with multimorbidity in the two boroughs. The term ‘multimorbidity’ is in increasing used in research and policy fields, but not necessarily in practice, so was not used explicitly in recruitment material. People who identified as ‘living with more than one long-term illness’ were recruited through leaflets displayed in a large community building where many charities and resource centres for those with long-term conditions were based, through a community newsletter, and through local peer-support networks for people with chronic diseases. The information sheet for key informants stated that the aim of the study was to map ‘services and support available to people living with long-term illness’ in order to ‘inform a larger study on multi-morbidity’. The topic guide began with an invitation to describe their own role, with follow-up questions using the terminology they used, and later questions prompting specifically on ‘multimorbidity’, if this was not a term already used by the interviewee. Interviews lasted between 45 minutes to over three hours and were, with consent, audio recorded and transcribed for coding and thematic analysis. In all fieldnotes, vignettes, and quotes, pseudonyms are used. Extracts are tagged with gender (M, F), and age for people living with multimorbidity, and KI (Key informant) number for policy and practice stakeholders (to preserve confidentiality in a relatively small field).

Analysis was primarily qualitative, using techniques of grounded theory (close reading of documentary and qualitative data, developing ‘codes’ which abstract common phenomena from those codes, and engaging in ‘constant comparison’ to develop logical inferences about the data and test emerging hypotheses) but orientated towards our aim of understanding what work the concept of multimorbidity was doing in this locality. This included a series of mapping exercises in which the actors, discourses and positions were listed (see Supplementary Material Table 1) and graphically linked to each other (see Supplementary Material Figure 1) to help reveal (for instance) what is core, what is missing, what positions are taken in the field. NVivo was used for quantitative documentary analysis (word co-occurrences). Ethics approval was granted by King’s College London (Ethics Approval Number: MRA-18/19-14,044 and HR-18/19-11,710).

## Multimorbidity reveals a fragmented system, in constant flux

A large number of actors – organisations, partnerships and initiatives – were identified, from the documentary analysis and interviews, as related to the management of multimorbidity in this field (see Supplementary Material, Table 1). The organisations operated at various levels, from international and national (funders, policy think tanks, government departments), regional (London-wide providers), to borough and local providers. They ranged in scope from specialist service-based organisations (such as those focused on housing or mental health needs) to large NHS trusts. They also ranged in organisational types, from voluntary organisations or social enterprises supporting people with one or more conditions (including faith-based organisations), to large statutory sector organisations. There was also a plethora of partnerships, alliances and cross-organisational bodies that linked these organisations. An immediately obvious characteristic of this field is that it is crowded. It was also widely described, by those within it – whether service users, clinical leads, commissioners or front-line practitioners – as ‘complex’. Understanding how organisational actors related to each other was not straightforward, as they are neither necessarily discrete, nor hierarchically organised (see Supplementary material, Figure 1). This is perhaps a typical late modern organisational field, with blurry boundaries around organisations, overlapping remits, multiple partnerships, and complex lines of accountability.

People living with multimorbidity typically described this health care system as difficult and often frustrating to navigate, evidenced in challenges such as cancelled appointments, missing notes and rapid turnover of staff and services:

… sometimes it doesn’t agree [between different systems] … this happened this year. … I went there [hospital] three times … [they said] I’m not in the system. I said I am in the system, I never changed, I should be there. When I attended there I’m not booked … You see me, I’m struggling. (P11, F, 66)

… my care coordinator, she left, again, this was the third time without, they don’t even bother to tell you when they’re leaving (P10, F, 38)

Service providers recognised these challenges for people with multiple conditions, and the limitations in integration across the separate services they may be accessing:

People with multiple long-term conditions often have an incredible hospital appointment burden, so will be seeing multiple specialists, often who don’t particularly talk to each other (KI1)

As one charity lead noted, health-oriented patient and voluntary associations are also typically organised around disease categories, even though ‘it is rare now to speak with anyone with only one condition’ (KI8). Even those with expertise and specialised knowledge of the field recognised that a comprehensive mapping of the organisations involved in service provision relevant to people with multimorbidity might not be possible. One manager of a service which aimed to provide navigation through services for clients with multiple health care needs suggested:

I think another problem is communication between services, you know […] Because I’m sure there might be more people specialised in housing advice but if the [connection service] doesn’t know it […] there are organisations as well, smaller ones that we are not using because we don’t know them (KI3)

The complexity of the local field is not, perhaps, surprising: people living with multiple conditions have multiple needs that cross healthcare specialisms and social services. Addressing the fragmentation of care was hardly a new challenge, and there had been, locally, a number of initiatives to better ‘integrate’ the system for patients whose needs might span a number of service providers. Multimorbidity might be the latest focus for thinking through system complexity, but it had not, in itself, revealed the problem.

Key informants drew on a number of related constructions of patients for whom system fragmentation might be a challenge, with ‘complex needs’ and ‘frailty’ often referenced. The overlaps between these concepts and multimorbidity were, at times, explicitly noted as confusing, and of course the most relevant framing might depend on the purpose:

with complex needs, there’s an agreed criteria on those with three or more long-term conditions and frailty (KI4)

[I]n a sense there’s a distinction here between … a group of people who have multiple long-term conditions, be they, you know, diabetes, kidney impairment, maybe they had a stroke, whatever, who are relatively … non-frail and then there’s a group of people who are frail who have multiple long-term conditions … from a medical point of view the care they need is quite different (KI1)

Asked what focusing on multimorbidity, specifically, had changed, one commissioning lead discussed practical initiatives (such as developing registers of patients with multiple conditions, so they could be identified more easily), but more strategically suggested the key change was a renewed impetus to commission services across sectors, as well as across different parts of the health care system:

It’s forced a lot more joint working programmes … and multidisciplinary working, and working with the voluntary sector … I think the multimorbidity work has gone along that route, with that general strategic difference [of] integration across different levels of health sector, across different sectors [KI2]

For this interviewee, multimorbidity had opened up both a more holistic approach, drawing in neighbourhood voluntary organisations, and potentially more ‘upstream’ work, by bringing in partners in local authorities responsible for prevention and wellbeing initiatives. Other participants echoed this possibility for a more preventative, upstream approach as being a change from previous iterations of partnership working. If a focus on ‘frail’ or ‘complex’ patients had suggested a primary orientation to clinical needs, then ‘multimorbidity’ implied an orientation to all those living with multiple conditions, not just patients. Emergent partnerships would, therefore, involve commissioning across local authority as well as health and voluntary sectors, and focus on prevention as well as disease management:

(The) main Health and Wellbeing Alliance, whatever they’re going to call it, is very much that concept of early disease and within an area having opportunities for that person to stay well. (KI1)

However, key informants also described a number of historical partnership initiatives and other strategic policies that had also been orientated to these kinds of cross-sector integration of services. What was striking was not only the rapidly proliferating number of organisational forms and varied initiatives that were currently aiming to deliver, commission or steer services for people now identified as having multiple morbidities, but also the large number of references to historical initiatives around partnership working for more holistic care for those who had previously been identified as having multiple or complex conditions. These extracts from one interview indicates this sense of both prior – and endlessly continuing – transformation of local organisational relationships. The interviewee references ‘Care Networks’, which had aimed at integrating services for clients identified as having ‘complex needs’; networks which ‘no longer exist’:

So there were three Local Care Networks in Lambeth and two in Southwark […] there were two parts to the Local Care Network […] a Local Care Network Board which brought together a variety of people from Primary, Secondary, Community Care and Social Care […] and then we had what we call the Local Care Network Forum which was really meant to be more patient facing, patient centred […]I suppose the big thing that came out of Local Care Networks in Lambeth was really a follow-on […] the Southwark and Lambeth Integrated Care Project (KI1)

These extracts suggest the relatively short time scales of many initiatives and partnerships to date, and the sense of a local system in constant flux. The Southwark & Lambeth Integrated Care partnership mentioned at the end of the extract above, for instance, reported in and operated until 2016 (only three years before our fieldwork), but some interviewees had not heard of it, and the report was no longer easily accessible. Partnerships were, it seems, always in the process of either emerging or dissolving:

So, the Local Care Networks were stood down once the PCN [primary care network] agenda came through, so the primary care and the community, or unit of function, is really the PCN, because obviously that’s the unit that’s nationally being driven. So, as the PCN Clinical Directors have come into place, we’re working at that, at that locality and that level … the LCN [local care network] footprint has really gone … so we’re sort of thinking that sort of the MDT [multi-disciplinary team] will be at a PCN level, whatever that looks like (KI4)

This permanent state of transformation was an ever-present challenge for those commissioning and delivering services within the system. One commissioning lead provided a detailed account of how orienting commissioning around multimorbidity would now entail an ‘alliance’ approach, commissioning across sectors. They also referenced several recent national health services policies that this commissioning work would have to align with as it emerged; summarising by exclaiming: ‘Oh, it’s insane! It’s insane, the pace of change’ (KI2).

This constant churn in initiatives, partnership structures, and staff in posts meant that recounting the history of organisational structures could be difficult, even for insiders, and recollecting past rationales, even more so. One interviewee, for instance, when asked about the development of one particular partnership, struggled to remember at which point discussions around how services fit together might have happened:

Well actually, there weren’t massive conversations about that. I think there would have been *[Pause]* I can’t recall any conversations specifically where we discussed that, which sounds a bit mad, and I’m sure they’re talking about it now (KI1)

## Addressing multimorbidity demands system transformation, not repair

The challenges people with multiple conditions faced in navigating a health care system designed around single morbidities were, then, widely recognised by professionals, and typically framed as a problem arising from ‘fragmented healthcare’. The solution was to (re)imagine pathways into the alternative: an integrated, holistic service provision.

For patients that’s very, very frustrating because they don’t perhaps understand the reasons why services don’t work better together. So a lot of this I think is about breaking down boundaries … (KI1)

There was broad agreement across all key informants that boundaries between specialities and community and hospital sectors were major barriers for patients, with the metaphors of ‘breaking boundaries’ and ‘silos’ ubiquitous. ‘Silos’ were framed as both the root of the problem *and* a continuing brake on improvement. Achieving integrated care therefore required ‘a massive shift in approach’ (KI8) to achieve a healthcare system geared to holistic and patient-centred care:

Acute and community health, it’s very siloed […] are you really providing them with a nurse for a truly holistic you know, wide-angled review […]? I think that that is the challenge with these complex patients, is how do we get everybody in that space to have that wide-angle lens? (KI4)

Traditional service provisions did not just act as a barrier through contract specifications for services, which pushed patients into speciality-focused disease care. They also undermined holistic care through technologies such as clinical guidelines, which were also disease-specific. Achieving patient-centred care for people with multimorbidity was therefore potentially in tension with clinical good practice as currently framed:

[the goal is] putting the person in the centre of all the decision making […] and on the opposite side of that and what’s stopping that is the disease guidelines. And you know particularly when you’ve got some with multiple morbidity, there can be 8 or 9 or 10 different disease guidelines that apply to them. (KI2)

This focus on the technologies of healthcare based on single diagnostic categories (such as separate clinics, or disease guidelines) as the cause of problems discursively positions fragmentation as an outcome of biomedical boundaries, not of the transformation itself. The challenges patients (and providers) face in the present are, then, firmly linked to the continued biomedical power of speciality ‘silos’ and traditional service boundaries, not to an emergent organisational field in constant flux. Whilst changing healthcare systems is undoubtedly a long-term and messy process, the rhetorical linking of the current challenge (fragmented care) to traditional barriers, rather than to current system complexity, did function as a rationale for continued (and perhaps always necessary) ‘transformation’.

A missing discourse, across the documents and from key stakeholders’ accounts, was that of system repair, or the restoration of what had been ‘lost’ as a result of greater specialisation. Roles which might once have provided an integrated, patient-focused service (such as GPs in the UK primary care model, who provided continuity and comprehensive care) were not evoked as models to return to, for instance. In our dataset, there were no nostalgic discourses of return. Overarching narratives were focused firmly on the disadvantages of ‘traditional’ health care (seen as the problem, not a solution) and on moving forward through transformation, rather than return or repair. Rather than calls for redressing the losses of disappearing generalist roles such as that of the GP or geriatrician, initiatives focused on either developing new roles that could ‘navigate’ a complex system on behalf of patients or their advocates, or initiatives which integrated specialties and care provision at the system level through alliances and partnerships. The few references to past strengths of the system were couched explicitly as belonging to a different world:

I suppose the world moved on a bit, where you’ve got the social prescribers and link workers sort of working to support patients you know, with the voluntary sector and sort of working with you know, the community at large. (KI4)

Multimorbidity was the latest lens through which the challenges faced by patients in navigating a health care system designed for diseases, not illness were made visible. Key informants suggested multimorbidity was different from previous framings of this challenge in its promise for a more holistic provision of services, across medical specialties and non-health care sectors. Multimorbidity was tightly framed as a problem of traditional speciality organisation (‘silos’); solutions were, discursively, therefore inevitably transformative, rather than restorative. However, the necessity for constant transformation across a health field still organised across specialities resulted in an ever-changing terrain of emergent and shifting partnerships, networks and initiatives in constant flux.

## Multimorbidity brings individual bodies, not relations, into focus

If the challenges patients face in accessing healthcare were linked discursively to the enduring limitations of biomedical categorisations, challenges in managing lives with multimorbidity were largely located as problems of individual bodies. In the content analysis of documents, we identified narratives that were centrally linked (in that they were frequently cited in relation to multimorbidity), including recurring themes: of ‘risks’ (324 mentions across all documents) or ‘risk factors’ (112 mentions) for multimorbidity; the idea of a patient ‘journey’ (mentioned 36 times across nine documents) through multimorbidity; and self-management. ‘Self-management’ was a widely referenced discourse through the documents, mentioned 24 times across eight documents, alongside ‘self-care’, used seven times in two documents.

Risk factors were primarily framed in professional discourses as sites of concern for their place on potential pathways or trajectories (‘gateway conditions’) to multimorbidity. The multiply-morbid body is framed as being on a single trajectory, sometimes a ‘journey’, through which the body declines over time, as further chronic illnesses are added (Ashworth et al., 2019). Within this journey, risk factors are thus potentially sites of intervention (or ‘windows of opportunity’), to avoid early and/or fast progression, and the ‘cumulative impact’ or ‘cumulative effects’ (mentioned seven times across four documents) of multiple long-term conditions. These risks are largely framed as belonging to individual patients or their ‘cultures’ rather than the structures which locate particular people in relation to others.

As has been widely reported in other studies (Porter et al., 2020), there were significant disjunctures between professional framings and accounts of lived experience, in that those living with multimorbidity rarely provided accounts of linear, unidirectional trajectories. That lived experience does not map to biomedical categories is perhaps not surprising. However, in terms of translating a desire for holistic understanding of patients’ perspectives into spaces for public health intervention, there is one key difference between professional and experiential framings that is noteworthy. That is the relationality of conditions that was evident in the accounts of people living with multiple conditions, but muted – or absent – in documentary and stakeholder accounts.

Relationality was salient in a number of respects in accounts of lived experience. First, it meant that the possibilities for maintaining health and wellbeing were deeply rooted in the social fabric. Relations with partners, families and friends (or their absences) were part of what made the ebb and flow of symptoms troublesome or manageable. This was particularly evident in accounts from those with caring responsibilities for others as well as their own chronic conditions to manage. Here, the interdependence of bodies disrupted the notion of the trajectory of multimorbidity relating to a single body:

… it does impact on me [caring for her husband], because I feel more pressured, and I feel as though, I need a bit of me as well […] I’m not saying that he doesn’t look out for me or look after me, he’s caring, yes, in his own way […] So sometimes I feel a bit lonely in that area, I want someone to do the same for me at times (P6, F, 71)

A second aspect of relationality was the ways in which, in lived experience accounts, morbidities (their emergence, their multiple and intertwined trajectories, and the significance and meanings of symptoms) were located in the relations of bodies within environments. Local environments (pollution, spaces to walk, availability of transport), working conditions, and financial resources, all shaped what it meant to have asthma, or arthritis, or mental health problems.

I think I can manage it okay, yeah. I always carry the inhaler with me and I can’t do anything about the traffic unfortunately but, yeah, that’s the way I manage it (P12, F, 79)

I’ve known from 50 years working in construction my joints are shot (P4, M, 71).

Front line providers also flagged the ways in which the health needs of those identified as having ‘multiple morbidities’ were located within these broader contexts. This lead for a service which signposted clients with ‘complex’ needs, noted:

But to be honest most of the clients’ needs are most the same, like it’s always about housing, it’s always about employment, it’s always about activities or counselling (KI3)

In contrast, these relational aspects of disease as rooted in social structures were relatively muted across policy documents and stakeholder interviews. Whilst key informants did note the possibilities that multimorbidity brought for commissioning across sectors, and addressing wellbeing for a healthy population as well as patients, as discussed above, these possibilities were not yet elaborated, and structural conditions were absent as an explicit driver of multimorbidity itself. Some policy documents did highlight structural conditions and the broader public sector context in this setting – an area of London characterised by stark wealth inequalities:

The recent welfare reforms, austerity measures and the economic downturn have affected disadvantaged communities the most. Making more affordable housing available and strengthening financial resilience are therefore priority actions to stop health inequalities from increasing further. (Document: Public Health Report for Lambeth 2013-14:6)

However, neither this backdrop of austerity nor the impact of structural inequality were articulated in direct connection with the problem of multimorbidity across key informant interviews or the policy documents. Austerity was the economic setting against which action had to be taken, or (on occasion) a potential lever for improvement – a ‘drive to work more efficiently’ (KI8) or more creatively. However, austerity was not framed as a problem that might contribute to the burden of multimorbidity for patients. In summary, inequalities in the distribution of multimorbidity were widely referred to; inequalities as possible causes of ill health were not.

## Discussion

In one setting, in south London, the relatively new concept of multimorbidity was a lens through which research, policy initiatives and service reorientations were being (re)focused. We undertook a situational analysis to explore what multimorbidity was doing, and with what implications for public health. We identified a field in a constant process of ‘transformation’. This was not new – a number of historical initiatives to address fragmented health care for patients variously described as being ‘frail’ or having ‘complex’ needs were described. Multimorbidity was, to an extent, simply the latest framing for service transformation to address the well-recognised challenges for particular groups of patients. However, the lens of multimorbidity was also described as offering novel promises. These were to a) reintegrate care towards whole persons, through integrating health services, and b) make possible a more ‘upstream’ approach by extending partnerships beyond health services. Addressing ‘multimorbidity’, specificially, provided a rational for system transformations that were explicitly premised on a critique of traditional biomedical nosologies, and the technologies and health care structures that arise from them. These had created ‘silos’, overly focused on the health needs of biomedical patients; multimorbidity would instead address whole persons. Discursively, positioning these silos as the primary driver of current problems undercut any possibility of system repair or restoration as a solution. In other settings, discourses of modernisation have been noted as co-existing with nostalgic discourses (McDonald et al., 2006); here, the emphasis was entirely on the need for radical transformation.

With its foundational categories of ‘disease’, modern medicine has long struggled with the loss of a holistic focus on ‘sickness’; the tensions between healing and curing continue to resound. Multimorbidity, as a concept, holds the promise of loosening these tensions, offering a biomedical lens through which whole persons, can be made visible to health providers. In this field, multimorbidity also held the promise of a resurgent public health approach, utilising data on population distributions of disease to identify the kinds of people and places where timely interventions can interrupt trajectories of decline, and commissioning across non-health sectors to address determinants of disease. However, our findings suggest some limitations in the realisation of this promise to date.

Considering multimorbidity as the result of discrete and multiple disease pathways produces a model of health that presupposes a linear journey located within one body; a journey that takes those with risk factors from developing one disease, then developing others, and then perhaps having to manage the effects of multiple treatment regimes. As much scholarship on living with multimorbidity has already suggested (Morris et al 201, Coventry et al., 2015), this metaphor of a journey from risks to disease fails to resonate with lived experience of chronicity, which may involve sudden disruptions, cyclical change, and ‘shifting perspectives’ (Paterson, 2001) throughout the temporalities of conditions in lives. We note here that it also fails to resonate with the more relational experience of living with multimorbidity, where disease is experienced as arising from the interplay of multiple bodies in environments over a lifecourse.

A situational analysis makes visible what is missing from the field, or less well connected between the actors. The lack of sustained attention to the structural drivers of multimorbidity is perhaps a surprising missing narrative in this field, given the focus of research on inequality, and the promise many participants noted of multimorbidity for providing a more public health approach to chronicity. The socially patterned nature of multimorbidity was well recognised: much epidemiological research focuses on inequalities in risk. However, the primary responses of the system here were around identifying individual patients on ‘trajectories’ and identifying the individual level risk factors that makes them vulnerable to developing a second and subsequent condition(s). Although people living with multiple conditions identified structural factors (pollution, manual labour), and service providers recognised the social determinants of health, the overwhelming emphasis of policy remained on individuals and lifestyles – reflecting the ‘lifestyle drift’ that has been widely documented elsewhere (Powell et al., 2017).

In current epidemiological research, multimorbidity constructs (inevitably) a ‘flattened’ version of ill health, in which diseases are added to each other, within a singular illness trajectory. This ‘flattening’ within one expansive illness category fails to address the well-documented experiences of living with multiple conditions, with their multiple trajectories of severity, importance, impact and concern. It also fails to incorporate the relational causes and consequences of multimorbidity. In focusing on quantity, through an additive approach to creating a new category of biomedical patient, multimorbidity remains inevitably situated as a property of bounded individual bodies, separate from their environment. Rather than seeing the multiply morbid body as a separate entity, the accounts of those living with multimorbidity evoke the relationality of bodies, conditions and environments in an interconnected, ongoing way. This opens up the body, not only as a category, but also in relation to change and the differing possibilities for intervention this may hold. Both categories of disease and the body are framed within the biomedical category of multimorbidity in ways that make it more difficult to see how structural aspects of health inequalities may contribute to living with, and developing multimorbidity. Ageing, deprivation, and marginalization are rendered as ‘risk factors’ that have the potential to impact individual bodies in which multimorbidity is ‘contained’, rather than properties of relational populations.

The relatively novel concept of multimorbidity is generating enthusiasm and focus as a helpful approach to addressing the problem of the growing and unequally distributed social and economic 'burden' of ill health', as well as the problems of health care fragmentation which were the major focus for key informants in this study. In one setting, we have identified how policy and practice actors leveraged the concept of multimorbidity as having the potential to reinvigorate holistic and upstream approaches to health and wellbeing in their locality. However, we suggest that multimorbidity, drawing on the logic of existing biomedical classification systems, inevitably replicates and reinforces the same problems with understanding and incorporating social and structural determinants of health. In this field, this was reflected in the challenges of addressing fragmented care through ever changing alliances and partnerships between organisations; and the need for constant transformation. In a biomedical system still founded on disease categories, these changes merely compounded complexity, rather than integrating an (inevitably) divided system. Our findings suggest that it is a different framing of disease and the body, rather than a new category, which is needed to push forward public health approaches to the challenges of chronic illness within populations.

## Supplementary Material

Supplemental Material
