# Supplementary material for: The (failed) promise of multimorbidity: chronicity, biomedical categories, and public health
Source: Crit Public Health. 2021 Dec 30;32(4):450–61. doi: 10.1080/09581596.2021.2017854 (PMC10461731; doi:10.1080/09581596.2021.2017854)

Table 1**: Initial map of actors in the field of multimorbidity in Lambeth & Southwark (from documents/policy interviews)**

**Table 1**	
**INDIVIDUAL HUMAN ELEMENTS/ACTORS**<br><br>Patient living with MM and family members<br>'Missing patients': those does not coded as having MM (due to proxy of prescription and/or use of GP Read codes underreporting)<br>'False positive' patients: those with non-chronic conditions (e.g. short term anxiety) who might not be coded as 'cured'<br>Carers<br>Specialists (rheumatologists, psychiatrists, and other specialists)<br>GPs<br>Doctors, Health care and service providers<br>Social workers; housing support workers; home care workers<br>Care coordinators: 'Integrated care manager(s)'<br>'wellbeing hub worker(s)'	**NONHUMAN ELEMENTS ACTORS/ACTANTS**<br><br>Classifications (GP Read codes, ICD 10, Frailty index; BP measures, mental health outcome instruments)<br>Data sources (HES; CPRD; Lambeth Data Net)<br>Technology platforms: GP electronic record system (EMIS)<br>KCL MM Spreadsheet of conditions<br>Virtual clinics; communications/phones/patient portals<br>Policies/initiatives – national NHSE Year of Care<br>NSFs & Clinical guidelines (as a barrier to care for patients)<br>QOF (as a source of 'bias' in terms of what is recorded on patient's conditions and diagnoses)
**COLLECTIVE HUMAN ELEMENTS/ACTORS**<br><br>NHS England; King's College Hospital NHS Foundation Trust; South London and Maudsley Foundation Trust; King's Health Partners; CCG(s); GP clinics; GP Federation(s); RCGP; Healthwatch local branches; SLIC Programme; Local Care Networks; Councils; Taskforce on Multiple LT Conditions (GST; Richmond Group of Charities; Royal College of General Practitioners (RCGP)); King's College London; Speciality clinics (St Georges diabetic foot clinic; Cardiac Clinics; Gastro clinics; Eating disorder clinic; GSST Cancer Centre; Psychologist and Psychologist clinics; therapists); Pay for service (massage, reflexologist); Community services: community nurses; community pharmacies; Community Mental Health Team (CMHT)); Community and Voluntary Orgs/activities (The Dragon Café; Support Groups: Prostate Cancer Support Group; Age UK Lambeth Safe and Independent Living (SAIL); Lambeth Integrated Reablement Service; self-management for life; diabetes patient forum; post-stroke care coordination; health huts; faith-based orgs)<br>Care Integration networks: Adults Local Services programme at GSTT; The Integrated Respiratory Team in Southwark and Lambeth; Health Innovation Network South London; Southwark and Lambeth Integrated Care 'Older People's Programme'; Lambeth Diabetes Care Team<br>Lambeth Together service delivery alliances: Neighbourhood & Wellbeing Alliance, Living Well Alliance.<br>Funders of activities: British Heart Foundation; GST; Population Health Management (PHM)<br>Research funders: MRC, GSST	**IMPLICATED/SILENT ACTORS/ACTANTS**<br><br>Those outside the NHS (non-patient living with MM)<br>Those does not coded due to proxy of prescription and/or use of GP Read codes); GP Register (underreporting; Read codes)<br>Travel/transport for MM<br>Sexuality and MM<br>Privacy concerns about data management<br>Financial cost to patient<br>Fee for service primary care/ prescriptions/counselling<br>Austerity<br>Read code replacements?<br><br>Lab tests; Blood; Body samples; Needles; Pharmaceutical technologies<br>Legitimate adult dependency<br>Mortality statistics<br><br>Empathy<br><br>Carer experiences of death and dying<br><br>Palliative care<br><br>London level/regional actors: GLA, Mayor's Health Inequalities Strategy, London Health Board

**DISCURSIVE CONSTRUCTIONS INDIVIDUAL AND/OR HUMAN COLLECTIVE ACTORS**<br><br>MM patients *as* complex patients with varied gradients of need: i.e. green = ‘LT conditions but is stable’; red is ‘severe level of disability/illness…high user of services’<br>MM as problem framed as pathways/journeys/trajectories:<br>1) Pathway into MM<br>• ‘acquisition sequence’; ‘cumulative impact’<br>• ‘gateway conditions’: Low SES (‘deprivation’ and ethnicity; ‘hotspots’; Hypertension; obesity; smoking as problem; mental health – particularly depression/anxiety (younger people); diabetes)<br>• Youth as site where MM has the potential to emerge<br>• Fast/early progression<br>2) Problem of ‘fragmented healthcare’ and need to create pathways into ‘integrated care’. Imagined future of integrated care:<br>• ‘Containing hospital admission’<br>• ‘Care coordination’<br>• ‘Holistic Care’<br>• Care Plans<br>• Tailored approach<br>• Emphasis on community health services; primary care in dialogue, or coordinated by a third party (a care coordinator) to create a ‘care coordination pathway’<br>3) MM as a problem of patients’ responsibilities<br>• Self-management; patient empowerment<br>Teams that involve the ‘patient and their carers’	**KEY EVENTS IN SITUATION**<br><br>AMS report published (2018)<br><br>Richmond Group Taskforce on Multiple Conditions report (Oct 2018)<br><br>Increasing concern about admissions to healthcare; polypharmacy; and ‘fragmented healthcare’<br><br>Multimorbidity funding availability (calls from MRC etc)<br><br>Access to data in Southwark and Lambeth<br><br>GGSTC jection of resources
**POLITICAL/ECONOMIC INTERESTS**<br><br>NHS financial interest – savings, drive for efficiency<br>Priority Area locally for: Richmond Group; GSTC Charity<br>Priority for national funders- availability of research funding	**DISCURSIVE CONSTRUCTIONS OF NONHUMAN ACTANTS**<br><br>Risk factors; Trajectories; journeys; ‘blurry boundaries’ of multimorbidity; Data ‘flows’ ; ‘shared care record’<br>Silos
**TEMPORAL ELEMENTS: UK HISTORICAL FRAME**<br><br>Ageing population; Histories of framing social deprivation as cause of health outcomes;<br>Health service reorganisations<br>NHS crisis and threat of being ‘overwhelmed’	**SOCIOCULTURAL/SYMBOLIC ELEMENTS**<br><br>Underpinning assumption – to be MM is to live with difficulties; idea of intervening at ‘windows of opportunity’
**MAJOR ISSUES/DEBATES**<br><br>Standardising definitions<br>Meaning of MM – what counts as MM [for project; local definitions; other research teams]<br>Distinguishing risk factors/conditions<br>Identifying the ‘burden’ from clusters	**SPATIAL ELEMENTS**<br><br>Bounded (LBs of) Southwark and Lambeth; neighbourhoods; neighbourhood-based care; ‘hotspots’; High ethnically diverse population (and specific MMs); urbanicity;<br>‘Silos’ of healthcare provision
	**RELATED DISCOURSES (HISTORICAL, NARRATIVE AND/OR VISUAL)**<br>Individualism/neoliberal discourses that places responsibility of healthcare on patient (‘self-management’); patient-centred/person-centred care

Figure 1 Example map of relationships between key actors: collective human actors and discursive elements

Example map of relationships between key actors

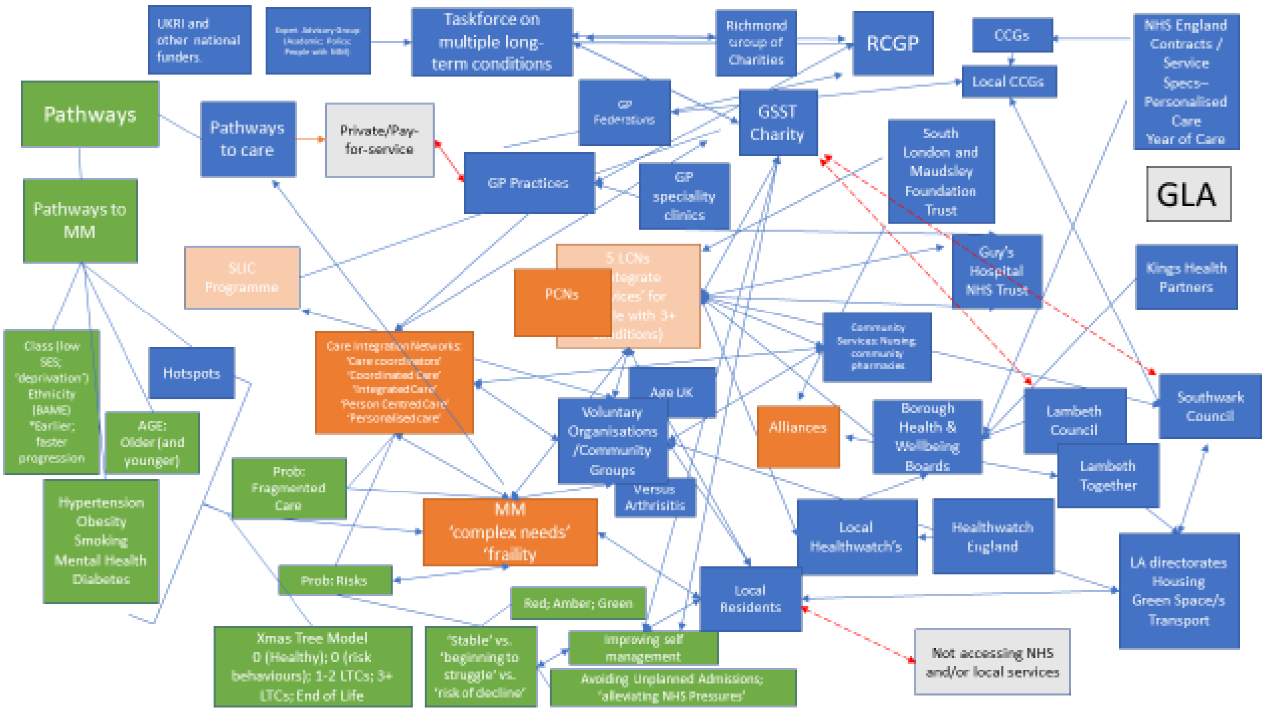

Supplement: Supplemental Material [file CCPH_A_2017854_SM7828.pdf]
